# Design of a Compact UHF Wilkinson Power Divider Using a Combined T-Shaped–CCMRC Resonator for Harmonic Suppression

**DOI:** 10.3390/mi17020158

**Published:** 2026-01-26

**Authors:** Saeed Roshani, Salah I. Yahya, Golshan Mohamadpour, Sobhan Roshani

**Affiliations:** 1 Department of Electrical Engineering, Ker.C., Islamic Azad University, Kermanshah 67189, Iran; 2 Department of Computer Technology Engineering, College of Technical Engineering, Al-Hadba University, Mosul 41002, Iraq; 3 Department of Electrical Engineering, Lorestan University, Khorramabad 68151, Iran

**Keywords:** compact resonator, filtering power divider, harmonic suppression, microstrip circuits, size reduction, UHF, Wilkinson power divider

## Abstract

This paper proposes a compact UHF microstrip divider with wideband harmonic suppression. A combined resonator, formed by a T-shaped resonator and a pair of coupled compact microstrip resonant cells (CCMRCs), is embedded into each divider branch to replace the conventional quarter-wavelength transmission lines. The divider is designed on an FR4 substrate (ε_r_ = 4.4, thickness = 60 mil) for a center frequency of 570 MHz. Full-wave electromagnetic simulations indicate equal power division at 570 MHz with return loss better than 39 dB and output-port isolation higher than 47 dB. Moreover, a wide stopband from 1.5 GHz to 3.5 GHz is obtained, yielding strong attenuation for the third-to-sixth harmonics. The proposed layout occupies 19.6 mm × 21.6 mm, which is about 76% smaller than a conventional 570 MHz divider (42.7 mm × 41 mm). The proposed design is suitable for modern wireless communication systems.

## 1. Introduction

Power dividers are fundamental building blocks in microwave and radiofrequency (RF) systems and are widely used in antenna arrays, balanced/combined power amplifiers, measurement setups, and phased-array front ends [1,2]. Among various configurations, the Wilkinson power divider is extensively used due to its excellent impedance matching, good isolation between output ports, and relatively simple planar structure [3]. Despite these advantages, conventional Wilkinson power dividers are inherently narrowband and exhibit limited suppression of higher-order harmonics, which can significantly degrade system performance in modern broadband and multi-standard wireless systems.

In modern wireless transceivers, undesired harmonics and spurious responses can increase in-band and out-of-band interference, cause spectral regrowth, and reduce the efficiency of active stages such as power amplifiers [4]. Therefore, embedding harmonic suppression directly into passive components (e.g., power dividers) helps simplify the RF chain and improves electromagnetic compatibility without adding extra filtering blocks. As a result, the design of compact power dividers with harmonic suppression has become an important research topic in recent years. Several techniques have been reported in the literature to mitigate harmonic effects, including the use of electromagnetic bandgap (EBG) structures [5], defected ground structures (DGS) [6], lumped-element loading [7], and coupled-line-based filtering networks [8]. Although these approaches are effective, they often suffer from increased design complexity, fabrication difficulty, or additional insertion loss.

Resonator-based filtering power dividers have emerged as a promising solution due to their ability to introduce transmission zeros at specific frequencies while maintaining a compact footprint [9,10,11,12,13,14].

In particular, microstrip resonators such as T-shaped resonators, compact microstrip resonant cells, and patch resonators have attracted considerable attention owing to their planar geometry, low fabrication cost, and compatibility with standard printed circuit board (PCB) technologies [15,16,17]. Patch-based resonators are especially attractive for UHF and microwave applications, where size reduction and wide stopband characteristics are critical design requirements.

Recent studies have demonstrated that integrating resonators directly into the branches of a Wilkinson power divider can simultaneously achieve size miniaturization and harmonic suppression without significantly degrading the fundamental operating performance [18,19,20,21]. However, many existing designs still suffer from either limited stopband bandwidth or insufficient suppression of higher-order harmonics. Moreover, achieving these features at UHF frequencies remains challenging due to the relatively large electrical size of conventional transmission-line-based structures.

In this work, these challenges are addressed by integrating a combined resonator into the branches of a Wilkinson divider. In this paper, a compact UHF power divider with wideband harmonic suppression is proposed. The designed divider is based on the integration of compact resonator structures, including a T-shaped resonator and two coupled CMRCs (CCMRCs), into the divider branches. By properly combining these resonators, multiple transmission zeros are generated, resulting in a wide stopband and effective wide suppression band. In addition, significant size reduction is achieved compared to a conventional Wilkinson power divider operating at the same frequency. The main contributions of this paper are the design of a compact combined resonator based on a T-shaped structure and a pair of coupled CMRCs, the development of a UHF Wilkinson power divider operating at 570 MHz with a wide suppression band from 1.5 GHz to 3.5 GHz that suppresses the third-to-sixth harmonics, and a significant size reduction. The overall size is 19.6 mm × 21.6 mm, which is about 76% smaller than a conventional 570 MHz power divider.

The remainder of the paper is organized as follows: Section 2 presents the resonator structures and explains how multiple transmission zeros are obtained. Section 3 and Section 4 describes the conventional baseline divider and the proposed compact topology, followed by discussions of S-parameters and performance metrics. Finally, Section 5 concludes the paper and outlines potential applications.

## 2. Resonator Design

This section studies different resonator designs. First, a T-shaped microstrip resonator is analyzed. A coupled compact microstrip resonant cell (CCMRC) is also investigated. These two structures are then combined to form a single resonator. The T-shaped resonator creates a transmission zero near the beginning of the stopband. The CCMRC produces two transmission zeros, one near the start of the stopband and another at higher frequencies. When these resonators are combined, several attenuation poles are formed. This leads to a wide stopband. The proposed resonator can later be used in the branches of the power divider.

**Figure 1:**
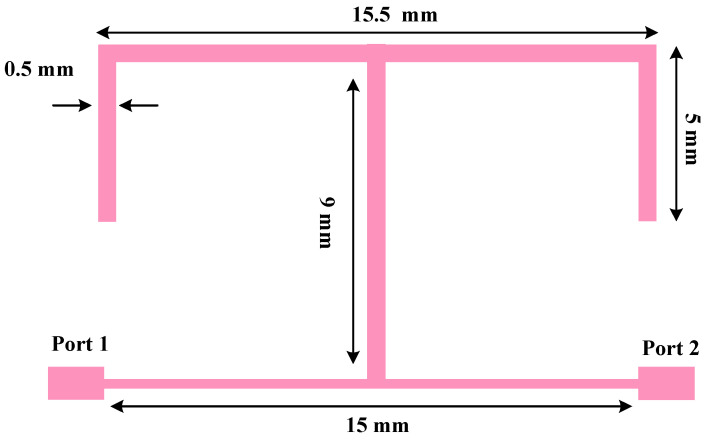
Geometry of the T-shaped resonator.

Figure 1 depicts the geometry of the T-shaped resonator designed based on an FR4 substrate (ε_r_ = 4.4, thickness = 62 mil).

**Figure 2:**
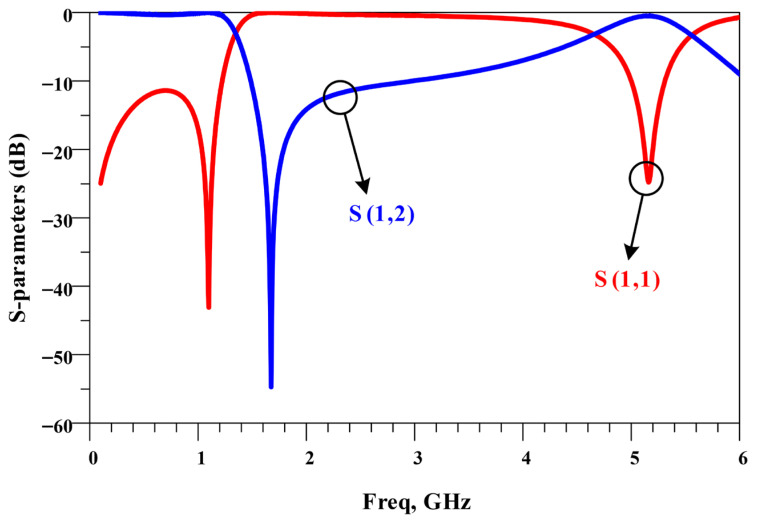
Frequency response of the T-shaped resonator with a transmission zero.

The simulated frequency response of the T-shaped resonator is shown in Figure 2. As indicated by the results, this simple resonator produces a single transmission zero near 1.6 GHz, where the magnitude of S21 sharply drops due to resonance. In this design, the location of the transmission zero can be easily tuned. When the stub length is increased, the electrical length becomes longer, and the transmission zero shifts to a lower frequency. In contrast, reducing the stub length moves the notch to a higher frequency.

**Figure 3:**
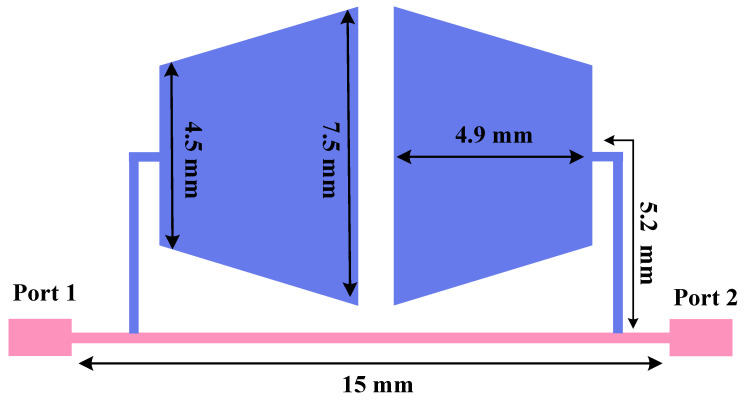
Geometry of the CCMRC resonator.

Figure 3 illustrates the CCMRC structure, where two compact trapezoidal microstrip sections are placed in a closely coupled configuration. The electromagnetic coupling between the adjacent sections enables multiple resonant modes in a small area. From a filter-design viewpoint, each mode can introduce an attenuation pole (transmission zero) in the stopband region, which helps suppress higher-order harmonics more effectively than a single resonator.

The applied CCMRC uses close proximity coupling to excite multiple resonant modes. Because the structure is compact, it is particularly suitable for UHF miniaturization where quarter-wavelength resonators would otherwise be physically large.

**Figure 4:**
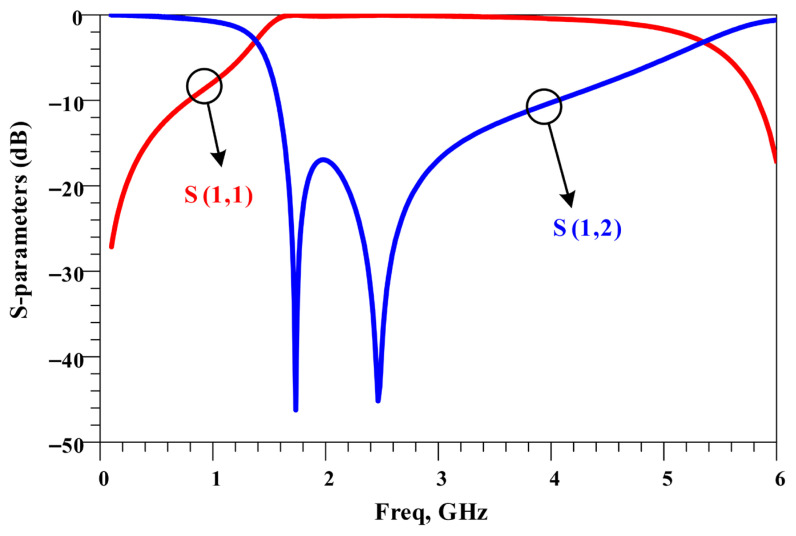
Frequency response of the CCMRC resonator with two transmission zeros.

The frequency response of the CCMRC resonator is presented in Figure 4. The CCMRC produces two transmission zeros at 1.8 GHz and 2.4 GHz, which are useful for extending harmonic suppression. In the CCMRC resonator, the location of the transmission zeros can be tuned. Increasing the coupling (reducing the gap) generally strengthens the notch depth and can slightly shift the resonance frequencies; loosening the coupling tends to weaken the attenuation and may narrow the effective stopband.

The two transmission zeros at 1.8 GHz and 2.4 GHz work together with the 1.6 GHz notch from the T-shaped resonator to widen the overall suppression region. A wide stopband is typically verified by checking that S21 remains below a chosen attenuation threshold over the full target band.

**Figure 5:**
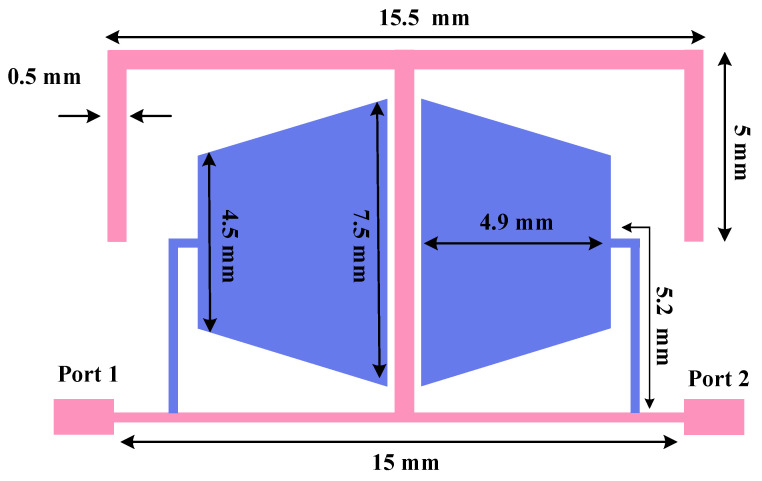
Geometry of the proposed resonator.

To obtain a broader stopband, the T-shaped resonator is combined with a CCMRC element, forming the proposed composite resonator in Figure 5. This combined structure is designed so that the individual transmission zeros of the sub-resonators are distributed across the different harmonic regions, thereby creating a continuous, wide suppression band rather than isolated notches.

The combined resonator can be viewed as a compact filtering section. When integrated into the divider branches, it simultaneously shortens the required electrical path and introduces out-of-band attenuation poles, which is the key mechanism behind size reduction and harmonic suppression in this work.

**Figure 6:**
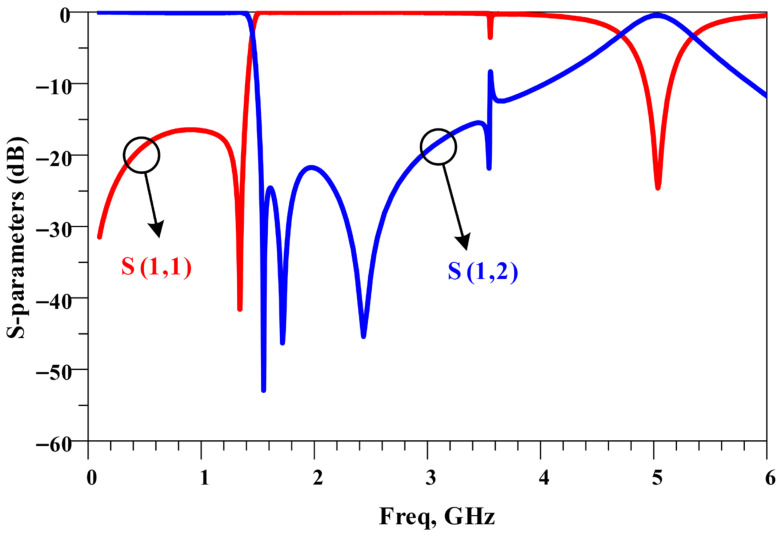
Frequency response of the proposed resonator with three transmission zeros and a wide stop band.

Figure 6 shows the frequency response of the proposed combined resonator. Three transmission zeros are generated near 1.6 GHz, 1.8 GHz, and 2.4 GHz, and a wide stopband bandwidth from 1.5 GHz to 3.5 GHz is achieved. In the context of a power divider, these zeros help suppress the third-to-sixth harmonics while minimally affecting the fundamental passband around 570 MHz.

Owing to its compact size and wide stopband behavior, the proposed resonator can be embedded into the quarter-wave branches of a Wilkinson power divider. This integration allows the divider to retain equal-split performance at the center frequency while introducing strong out-of-band attenuation, which is highly desirable for UHF front ends that operate close to stringent spectral masks.

**Figure 7:**
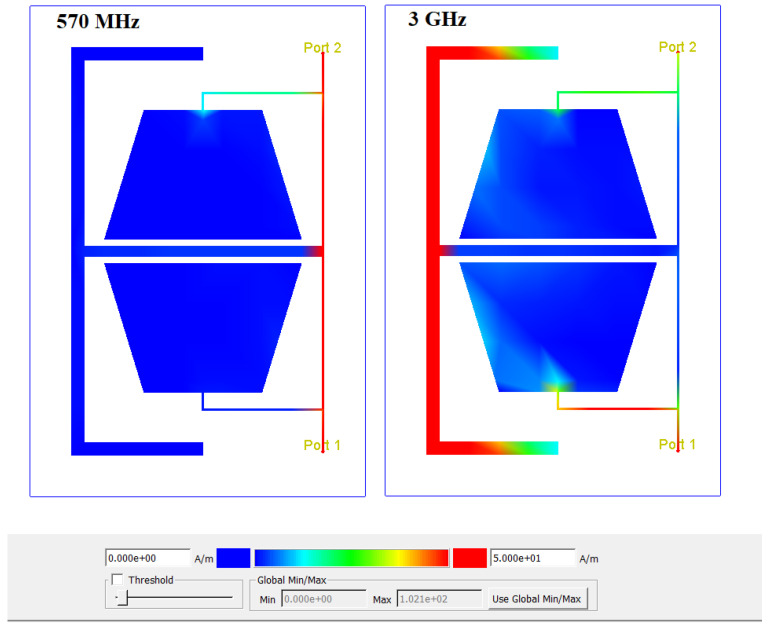
Current distributions of the proposed resonator at two different frequencies: 570 MHz, which lies in the passband, and 3 GHz, which is located in the stopband.

Figure 7 shows the current distribution of the proposed resonator at two frequencies, 570 MHz and 3 GHz. At 570 MHz, which is inside the passband, the input signal flows smoothly from port one to port two. In contrast, at 3 GHz, which lies in the stopband, the proposed resonator blocks the signal, and no current reaches port two, which clearly demonstrates the correct performance of the proposed resonator.

## 3. Proposed UHF Power Divider

Both the conventional and the proposed power dividers are implemented on an FR4 substrate with relative permittivity ε_r_ = 4.4 and a thickness of 62 mil. A 50 Ω system impedance is assumed. For reference, a conventional equal-split Wilkinson divider employs two quarter-wavelength branches with a characteristic impedance of 70.7 Ω and an isolation resistor of 100 Ω at the design frequency. At 570 MHz, these quarter-wavelength lines occupy a large physical length on FR4, which motivates the use of compact resonator-based replacements.

With the chosen substrate, the electrical wavelength (λ) at 570 MHz is 288 mm.

**Figure 8:**
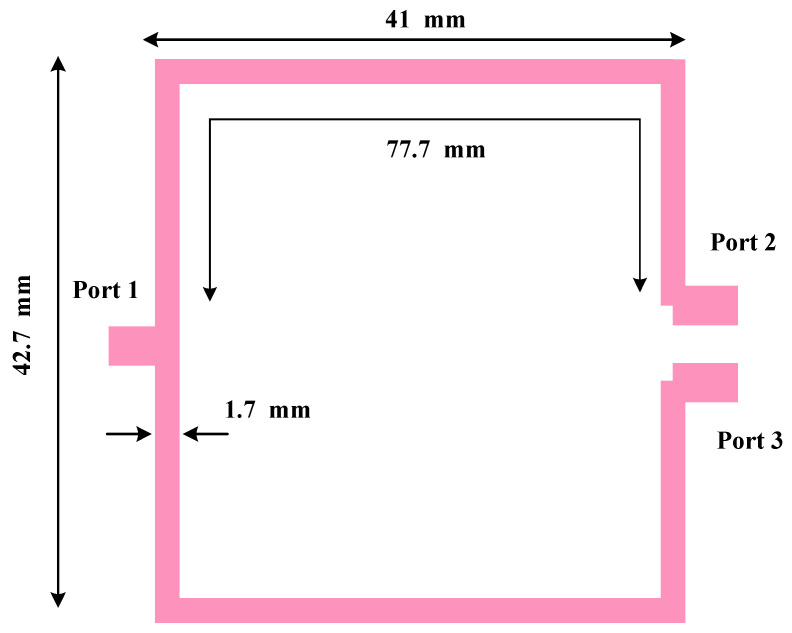
Geometry of the conventional UHF power divider with an operating frequency of 570 MHz.

The layout of the conventional UHF Wilkinson power divider designed for 570 MHz is shown in Figure 8. While the conventional structure is straightforward to design and fabricate, it occupies a relatively large area of 42.7 mm × 41 mm because the branch lines must be approximately a quarter-guided wavelength at the operating frequency.

In the even mode, ports two and three are in phase, so the isolation resistor carries no current (it is open in even mode). Each output port is terminated with *Z*_0_ = 50 Ω. Each branch is a λ/4 line with characteristic impedance $Z_{\mathrm{branch}}$. This line transforms the load as follows:

(1) $$Z_{\mathrm{in},1}=\frac{Z_{\mathrm{branch}}^{2}}{Z_{0}}$$

At the input port, the two identical branches are in parallel, so the total even-mode input impedance is as follows:

(2) $$Z_{\mathrm{in},\mathrm{even}}=\frac{Z_{\mathrm{in},1}}{2}=\frac{Z_{\mathrm{branch}}^{2}}{2Z_{0}}$$

For perfect input matching, $Z_{\mathrm{in},\;\mathrm{even}}=Z_{0}$. In this case,

(3) $$Z_{0}=\frac{Z_{\mathrm{branch}}^{2}}{2Z_{0}}\Rightarrow Z_{\mathrm{branch}}=\sqrt{2}\;Z_{0}=70.7\;Ω\;$$

**Figure 9:**
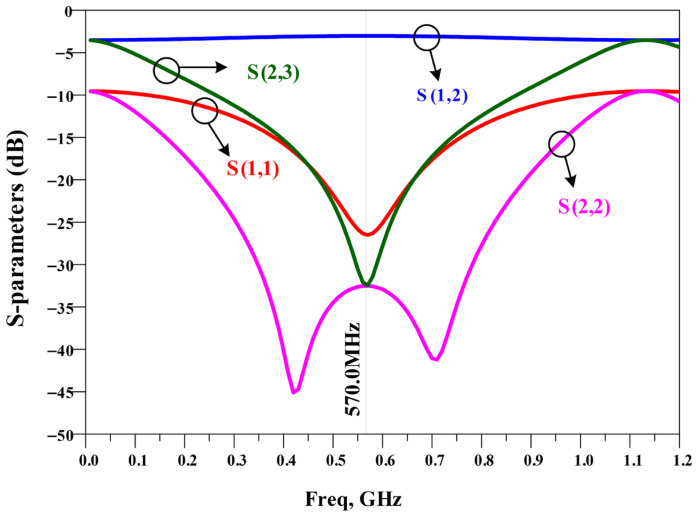
Frequency response of the conventional UHF power divider with an operating frequency of 570 MHz.

The simulated S-parameter response of the conventional divider is presented in Figure 9. The divider operates correctly at 570 MHz with the expected equal split; however, because it is composed mainly of transmission-line sections, it does not inherently suppress spurious higher-order responses. Consequently, unwanted harmonics can pass through the network at higher frequencies, which is undesirable in broadband RF front ends.

**Figure 10:**
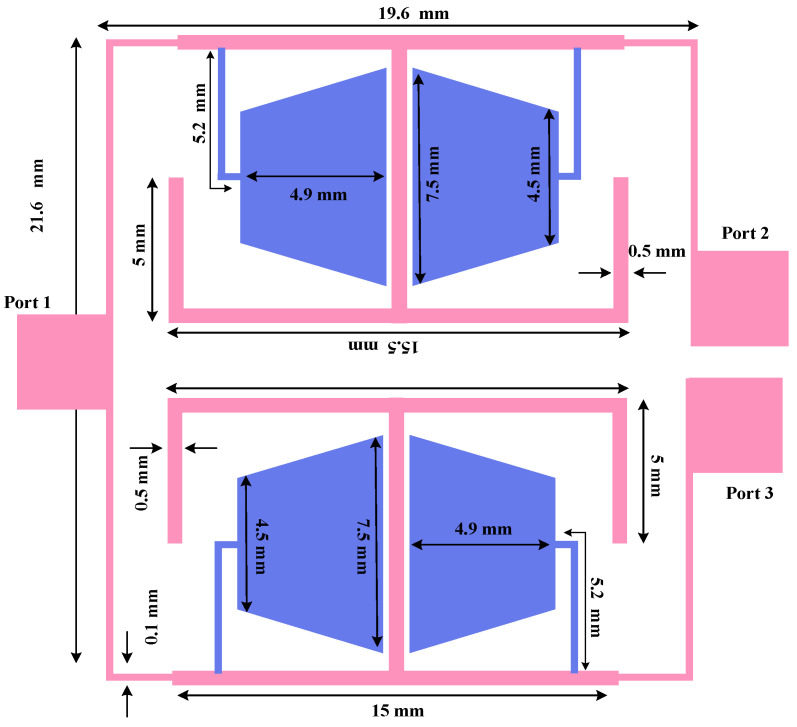
Geometry of the proposed UHF power divider with an operating frequency of 570 MHz.

To overcome the large footprint and the lack of harmonic rejection in the conventional design, the proposed UHF divider is developed as illustrated in Figure 10. In the proposed topology, the combined resonators described in Section 2 are embedded into the main divider branches to replace the conventional quarter-wavelength transmission lines. This modification achieves approximately 76% size reduction while introducing a wide stopband for harmonic suppression. The resulting compact layout occupies an area of 19.6 mm × 21.6 mm. Based on the electrical wavelength (λ) of 288 mm at 570 MHz, this size corresponds to 0.06λ × 0.07λ.

**Figure 11:**
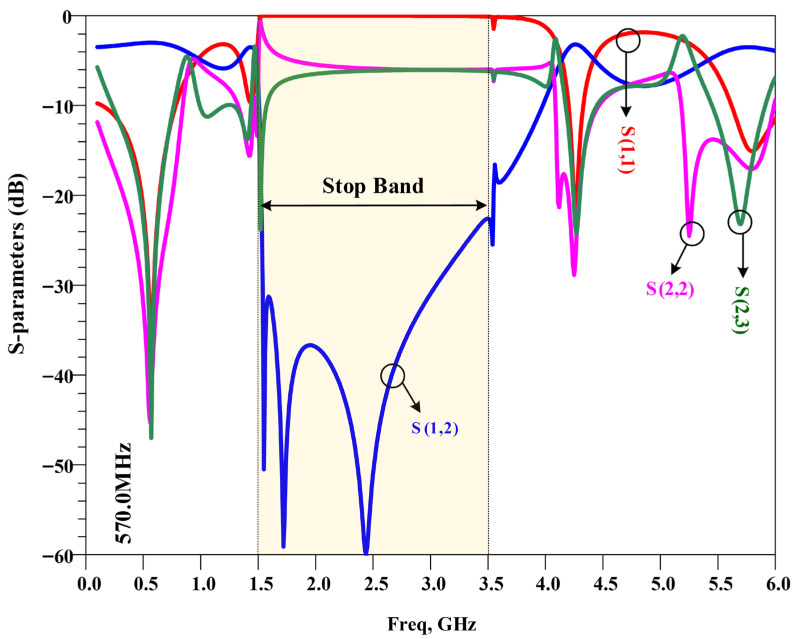
The simulation frequency response of the proposed UHF power divider with an operating frequency of 570 MHz.

Full-wave electromagnetic simulations were carried out to validate the performance of the proposed power divider, including matching, isolation, insertion loss, and out-of-band suppression. The simulated frequency response is shown in Figure 11. At the operating frequency of 570 MHz, the divider exhibits good impedance matching at the input and an equal in-phase split between the two outputs (transmission close to −3 dB per branch). The return loss is better than 39 dB, and the isolation between the output ports exceeds 47 dB, confirming proper Wilkinson operation. In addition, strong attenuation is observed from 1.5 GHz to 3.5 GHz, which demonstrates effective suppression of the third-to-sixth harmonics by the embedded resonators. These characteristics make the proposed divider suitable for compact UHF wireless subsystems where both size and spectral cleanliness are important.

It should be noted that, in a three-port power divider, good performance at the center frequency is typically confirmed by a low |S11| value, indicating good impedance matching, nearly equal |S21| and |S31| values, indicating equal power division, and a high |S23| value, indicating good isolation between the output ports. For harmonic suppression, an important requirement is that |S21| and |S31| remain highly attenuated over the specified stopband from 1.5 GHz to 3.5 GHz. The proposed power divider satisfies all these conditions and demonstrates very good overall performance.

**Figure 12:**
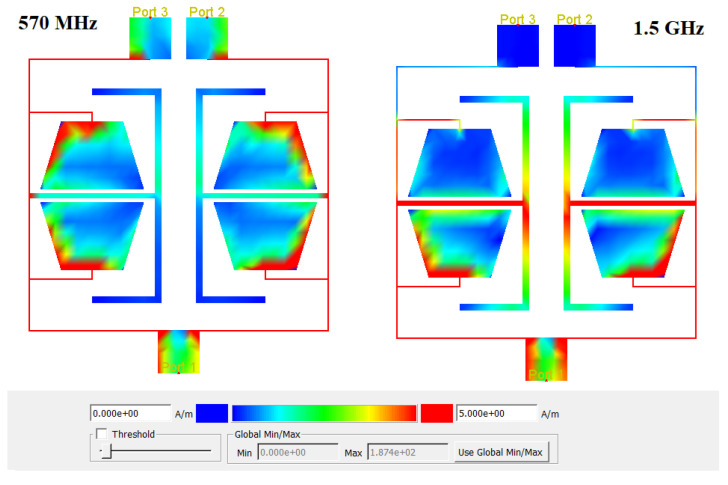
Current distributions of the proposed UHF divider at two different frequencies: 570 MHz, which lies in the operating frequency, and 1.5 GHz, which is located in the rejection band.

Figure 12 shows the current distribution of the proposed three-port UHF power divider at two frequencies, 570 MHz and 1.5 GHz. At 570 MHz, which is the operating frequency, the input signal enters through port one and is equally divided between ports two and three. In contrast, at 1.5 GHz, which lies in the rejection band, the divider suppresses the signal, and almost no current reaches ports two and three. These results clearly demonstrate the correct operation and good performance of the proposed UHF power divider.

**Figure 13:**
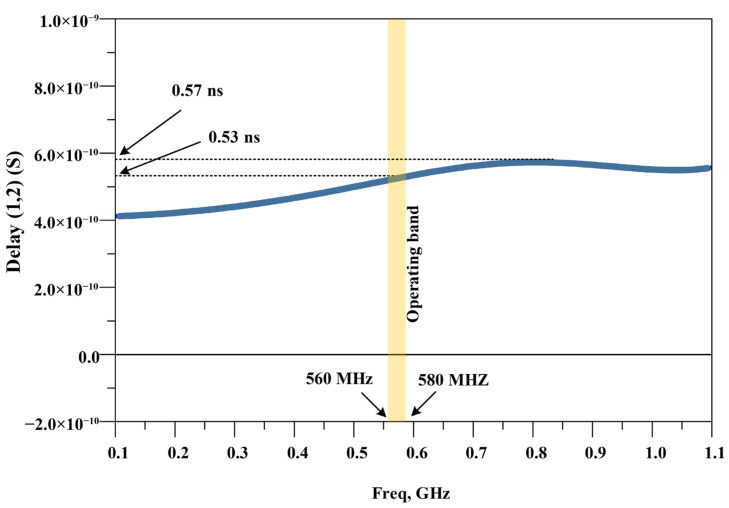
Group delay of $S_{21}$ for the proposed UHF power divider from 0.1 to 1.1 GHz (the operating band of 560–580 MHz is highlighted).

Figure 13 presents the group delay of $S_{21}$ for the proposed divider. The group delay is computed from the phase response of $S_{21}$ as $\tau_{g}=-d\phi /d\omega$. In the operating band of 560–580 MHz, the group delay is very flat. The maximum group delay in this band is less than 0.53 ns. The peak-to-peak variation in this band is about 0.02 ns (≈20 ps). This small ripple shows good phase linearity in the operating band. Therefore, the divider can preserve signal shape and is suitable for digital modulation at UHF. Over the wider range of 0.1–1.1 GHz, the maximum group delay remains below 0.57 ns, which confirms stable wideband phase behavior.

## 4. Fabrication and Measurements

**Figure 14:**
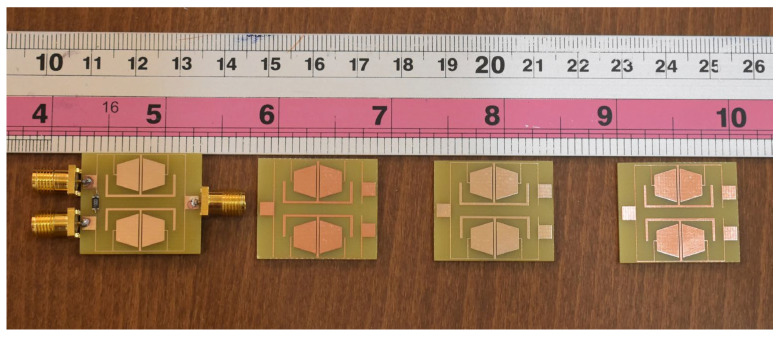
Implemented prototypes of the proposed UHF power divider based on the FR4 substrate.

The proposed power divider is fabricated on the FR4 substrate with relative permittivity ε_r_ = 4.4 and a thickness of 62 mil. Several implemented prototypes of the proposed UHF power divider are depicted in Figure 14.

**Figure 15:**
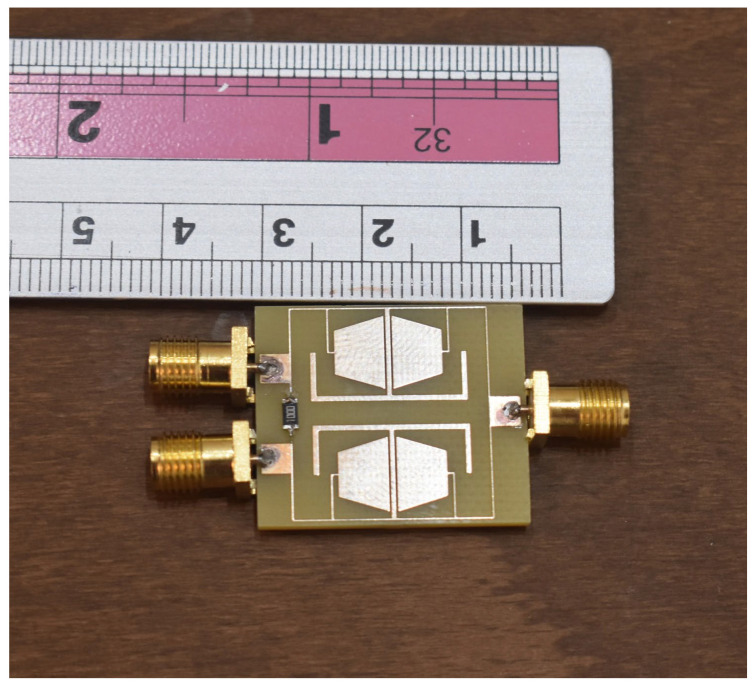
The implemented photo of the proposed UHF power divider on the FR4 substrate.

According to the fabricated limitations and tolerances in the implemented device, there are a few changes in the simulated results and implemented prototypes. Therefore, to minimize these effects, several prototypes are fabricated and tested. The device with the best performance is chosen as the proposed device. The implemented photo of the proposed UHF power divider is depicted in Figure 15.

**Figure 16:**
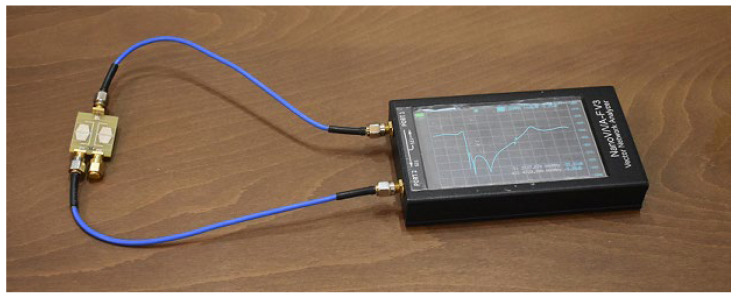
Measurement setup and device under test during measuring the S_21_ parameter of the proposed UHF divider with VNA.

The Nano VNA-FV3 vector network analyzer (Hangzhou SYSJOINT Information Technology Co., Hangzhou, China) is used to measure the S-parameters of the proposed UHF divider. The measurement setup and device under test during measuring the S_21_ parameter of the proposed UHF divider are depicted in Figure 16.

**Figure 17:**
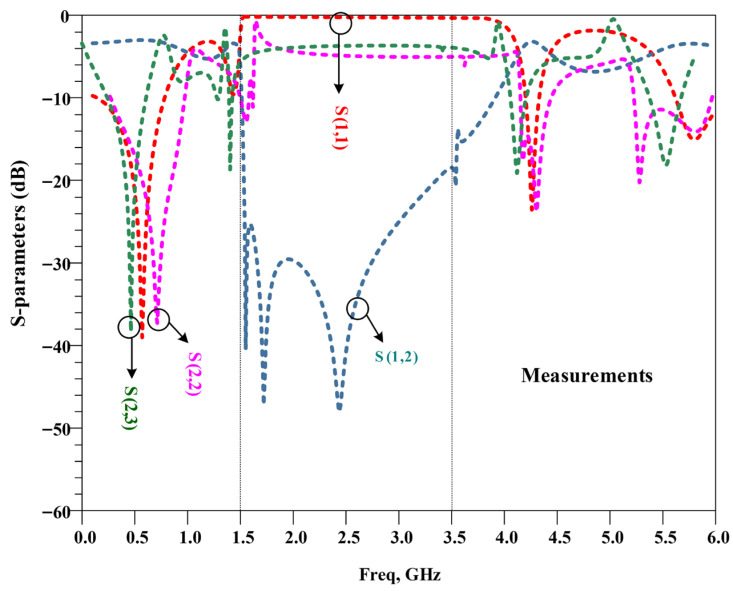
The measurement frequency response of the proposed UHF power divider with operating frequency of 570 MHz.

The measurement frequency response of the proposed UHF power divider is depicted in Figure 17. As the results show that the measured S-parameters are in good agreement with simulation parameters, the measured results validate simulations and design performance.

A performance comparison between the proposed UHF divider and representative related designs is summarized in Table 1. To facilitate a fair comparison, the table lists the center frequency, harmonic suppression levels, size reduction (S.R.), normalized circuit size (in terms of λg × λg), output-port isolation, insertion loss (IL), and return loss (RL). As seen in the final row, this work simultaneously achieves substantial size reduction (76%) and strong multi-harmonic suppression while maintaining high isolation (47 dB) and low insertion loss (0.1 dB).

The normalized size is expressed in guided-wavelength units (λg × λg) to allow fair comparison across different frequencies and substrates. For example, a smaller λg-normalized area generally indicates a more compact electromagnetic implementation, even if the absolute dimensions differ.

## 5. Conclusions

A compact UHF microstrip Wilkinson power divider with wideband harmonic suppression is presented. The proposed divider is designed to operate at 570 MHz on an FR4 substrate with a relative permittivity of 4.4 and a thickness of 62 mil. The design employs a compact combined resonator consisting of a T-shaped resonator and a CCMRC resonator. These resonators are integrated into the divider branches to improve performance.

By using this combined resonator structure, a significant size reduction is achieved while effectively suppressing higher-order harmonics. The overall size of the proposed divider is 19.6 mm × 21.6 mm, which is about 76% smaller than a conventional UHF Wilkinson divider. In addition, the divider provides a wide stopband from approximately 1.5 GHz to 3.5 GHz, enabling suppression of the third-to-sixth harmonics.

The results show high isolation between the output ports, exceeding 47 dB, and good impedance matching, with a return loss better than 39 dB at the center frequency. These results confirm stable operation and strong harmonic suppression. Due to its compact size, simple structure, and good performance, the proposed power divider is well-suited for modern UHF wireless communication systems where both size reduction and harmonic control are critical.

## Figures and Tables

**Table 1:** Performance comparison of the proposed UHF divider with related dividers.

Ref.	Center Freq. (GHz)	Substrate/Dielectric Constant	Harmonic Suppression (dB)					S.R. ^1^ (%)	Size (λg × λg ^2^)	Isolation (dB)	IL ^3^ (dB)	RL ^4^ (dB)
			2nd	3rd	4th	5th	6th					
[22]	2.55	Rogers RT-duroid 5880/2.2	20	20	23	24	32	43.5	N.R.	46	0.15	27
[23]	2.65	F4B/2.65	-	29	-	34	-	63.5	N.R. ^5^	22	0.4	27
[24]	2	Rogers RT-duroid 5880/2.2	-	53	25	56	20	29.3	N.R.	45	0.1	45
[25]	0.9	FR4/4.4	-	22	-	-	-	47	N.R.	N.R.	0.3	36
[26]	7	Al_2_O_3_/9.8	-	-	-	-	-	-	0.2 × 0.19	12	0.4	15
[27]	2.4	Rogers RT-duroid 5880/2.2	-	-	-	-	-	43.1	0.35 × 0.47	16	0.2	16
[28]	1	Rogers RO4003C/3.55	21	25	-	-	-	-	0.19 × 0.25	29	0.2	24
This work	0.57	FR4/4.4	-	56	48	38	26	76	0.07 × 0.06	47	0.1	39

^1^ Size reduction (S.R.) is reported as the percentage decrease in occupied area relative to a conventional Wilkinson divider at the same center frequency. ^2^ λg is defined as the guided wavelength on the microstrip line: $\lambda_{g}=\lambda_{0}/\sqrt{\varepsilon_{\mathrm{eff}}}=c/\left(f\sqrt{\varepsilon_{\mathrm{eff}}}\right)$. ^3^ Insertion loss (IL) corresponds to the additional loss beyond the ideal −3 dB split, usually evaluated at the center frequency. ^4^ Return loss (RL) indicates input matching performance at the center frequency (higher values imply better matching). ^5^ Not reported (N.R.) in the cited reference.

## Data Availability

All the data collected in the study are mentioned in the article.
